# Combining QTL mapping and RNA-Seq Unravels candidate genes for Alfalfa (*Medicago sativa* L.) leaf development

**DOI:** 10.1186/s12870-022-03864-7

**Published:** 2022-10-11

**Authors:** Xueqian Jiang, Xijiang Yang, Fan Zhang, Tianhui Yang, Changfu Yang, Fei He, Ting Gao, Chuan Wang, Qingchuan Yang, Zhen Wang, Junmei Kang

**Affiliations:** 1 Institute of Animal Science, Chinese Academy of Agricultural Sciences, Beijing, China; 2 Institute of Animal Science, Ningxia Academy of Agricultural and Forestry Sciences, Ningxia, China

**Keywords:** Alfalfa, Leaf development, QTL mapping, RNA-seq

## Abstract

**Background:**

Leaf size affects crop canopy morphology and photosynthetic efficiency, which can influence forage yield and quality. It is of great significance to mine the key genes controlling leaf development for breeding new alfalfa varieties. In this study, we mapped leaf length (LL), leaf width (LW), and leaf area (LA) in an F1 mapping population derived from a cultivar named ZhongmuNo.1 with larger leaf area and a landrace named Cangzhou with smaller leaf area.

**Results:**

This study showed that the larger LW was more conducive to increasing LA. A total of 24 significant quantitative trait loci (QTL) associated with leaf size were identified on both the paternal and maternal linkage maps. Among them, nine QTL explained about 11.50–22.45% phenotypic variation. RNA-seq analysis identified 2,443 leaf-specific genes and 3,770 differentially expressed genes. Combining QTL mapping, RNA-seq alalysis, and qRT-PCR, we identified seven candidate genes associated with leaf development in five major QTL regions.

**Conclusion:**

Our study will provide a theoretical basis for marker-assisted breeding and lay a foundation for further revealing molecular mechanism of leaf development in alfalfa.

**Supplementary Information:**

The online version contains supplementary material available at 10.1186/s12870-022-03864-7.

## Background

Photosynthesis has become a major goal for breeders aiming to further improve yields in field crops [1], which is directly affected plant growth and grain yield [2]. Leaves are the main photosynthetic organs in plants. It has been reported that leaf size has a significant effect on photosynthetic rate, transpiration, and carbon fixation, which contribute to plant biomass [3–5]. Therefore, it is feasible to produce new cultivars with high yield by optimizing the leaf-related traits of crop plants.

Given its obvious effect on plant growth, the genetic mechanism that control leaf size has been investigated intensively in model plants [6, 7]. The leaf size and growth are controlled by several regulators, including transcriptional factors, phytohormones and microRNAs [6]. In Arabidopsis, phytohormones (auxin, gibberellin, cytokinin, and brassinosteroid) biosynthesis or signaling genes have been cloned and identified in leaf area regulation [6]. For instance, the disturbance to auxin homeostasis and signaling achieved by over‐expressing *PID* resulted in plants which produce plants exhibiting smaller than normal leaves [8]. Meanwhile, QTL and candidate genes associated with leaf size have been reported in grass plant species, such as maize [9], rice [10, 11], wheat [12], and barley [13]. Using a recombinant inbred line population with a single-nucleotide polymorphism (SNP) genetic linkage map, a major QTL for leaf width of maize was further fine-mapped to an interval of 55 kb and two candidate genes were identified in developing leaves [14]. In rice, enhanced expression of a QTL related to leaf length, *qLL9*, significantly increased the grain size, thousand-grain weight, and grain yield [15]. Also, legumes, such as soybean [16], common bean [17], and peanuts [18] have been investigated for the genetic basis of variation in leaf size. In pea, 37 bulked segregant analysis markers associated with leaf traits were identified by using QTL mapping combined with BSA [19].

Alfalfa (*Medicago sativa* L.), a perennial forage legume, has been cultivated for hay, pasture, and silage worldwide [20, 21]. One of the primary goals of alfalfa breeding programs is to produce new cultivars with high biomass, as the primary commercial determinant of alfalfa production is its above-ground biomass. Therefore, it is feasible to improve photosynthetic efficiency and yield by optimizing the leaf-related traits of alfalfa.

Autotetraploid alfalfa, a self-incompatibly cross-pollinated crop, have a very complex genome [22]. This complex genetic nature, eg the complex loci segregate, hinders the construction of genetic linkage maps to improve important agricultural traits in alfalfa. The strategy of using limited biallelic markers segregating in specific patterns are suitable for constructing genetic linkage maps of the tetraploid crop. Single dose allele (SDA) markers unique to each parent with segregation of 1:1 (Aaaa x aaaa) can be used to construct genetic linkage maps for an F1 populations, which was derived from two heterozygous alfalfa genotypes [23]. As such, JoinMap software, which was designed for diploid species, were used to construct linkage maps in tetraploid alfalfa [23–25]. Also, there have been reported on alfalfa QTL associated with important agronomic traits, including biomass-related traits [25, 26], spring vigor [27], as well as stress-related traits including winter hardness [28], and leaf rust resistance [29].

The genetic and genomic basis of leaf size has been investigated extensively in cereals crops, whereas there is scarce on herbaceous perennials, such as alfalfa. Although a number of QTL have been identified in alfalfa, the genetic architecture and main-effect QTL/genes related to leaf development remain unclear. Integration of RNA-seq and QTL mapping had been considered to be a reliable method to rapidly identify potential candidate genes. Although it has been applied in rice and maize [30, 31]. The strategy has not been reported in alfalfa leaf development related genes. The objectives of this study were: (1) to identify QTL associated with alfalfa leaf development, and (2) to locate potential candidate genes according to QTL mapping, differentially expressed genes (DEGs) analysis, and functional annotation. The identified QTL and potential candidate genes would be helpful for alfalfa breeding program.

## Materials and methods

### Mapping population

Mapping population and Field location were as described previously [32]. Briefly, the the *Medicago*. *sativa* subsp. *sativa* landrace (Cangzhou) with smaller leaf area was used as paternal parent (P1), and the *Medicago*. *sativa* subsp. *sativa* cultivar (ZhongmuNo.1) with larger leaves was used as maternal parent (P2). In 2018, 388 of the original progenies (F1 population) generated by crossing survived and under phenotypic investigation for this study. Both the parents and the F1 were grown at two locations; Langfang (LF), Hebei Province, China (39.59°N, 116.59°E) and Changping (CP), Beijing, China (40.18°N, 116.24°E), using a randomized complete block design with three replications.

### Phenotypic data collection and analysis

Phenotypic data were collected at the two locations (CP and LF) in the year of 2018 (CP18 and LF18) and 2019 (CP19 and LF19). In order to ensure the consistency of sampling at different locations in different years, leaves of all genotypes were sampled at the initial flowering stage (the first flower appeared). The leaf from the middle leaflet of the third or fourth fully expanded trifoliate leaf from the shoot tip of each plant were chosen for sampling. For each plant, three leaves were sampled. Leaf size related phenotype, leaf length (LL), leaf width (LW), and leaf area (LA) were measured using a LAM-B type handheld leaf area meter (Shijiazhuang Fansheng Technology Co.; Ltd). Best linear unbiased predictions (BLUPs) were analyzed of the phenotypic data using PROC MIXED [33]. Interaction of a given genotype with different environments was estimated using PROC GLM (SAS Institute, 2010). Correlation of leaf-related traits was analyzed using the R package R/corrplot. The broad-sense heritability (H^2^) was calculated using the AOV (Analysis of variance) function of QTL IciMapping [34].

### Genetic linkage map construction and QTL mapping

The methods used for linkage map construction and QTL mapping were described previously [32]. Briefly, DNA samples of the progenies and both parents were extracted according to the manufacturer’s protocol from ~ 100 mg of fresh leaf tissues using the CWBIO Plant Genomic DNA Kit (CoWin Biosciences, Beijing, China). Single dose allele (SDA) markers with a segregation ratio of less than 2:1 among F1 progenies were used to construct a genetic linkage map using JoinMap 4.0 according to a method published previously [23].

QTL detection was performed by inclusive composite interval mapping (ICIM) using the QTL IciMapping Software (version 4.1) [34, 35]. For the phenotypic data in single environment and the BLUP value, QTL were analyzed using the ICIM-ADD mapping method with the default parameters (PIN = 0.001, Walking step = 1.0 cM, type I error = 0.05), and the significance threshold values (*P* ≤ 0.05) were determined through 1000 permutation tests. The result of QTL analysis was obtained and imported to the software MapChart to display results [36].

### RNA-seq data analysis

In previous study, RNA-Seq project of leaves of two different *Medicago sativa* genotype (*Medicago sativa* subsp. *sativa* cv. Beaver and *M*. *sativa* subsp. *falcata* accession PI 641,381) was performed [37]. Significant difference was noted between genotypes in terms of leaf size, while they had no difference in plant height and biomass. The first expanded leaflet was harvested from four biological replicates of ‘*sativa*’ and ‘*falcata*’, respectively. Also, a RNA-seq project of alfalfa different tissues (leaves, roots, nodules, flowers, elongating internodes, and post-elongation internodes) was performed in previous [38]. Leaves were harvested 28 days after the second cutback. In this study, to identify leaf-specific expression genes and DEGs between different leaf size genotypes, the two RNA-seq projects were download. RNA-Seq data quality was estimated by FastQC v0.11.9 (http://bioinformatics.bbsrc.ac.uk/projects/fastqc), and low quality sequences were filtered using fastp v0.12.4 [39]. Then, clean reads were mapped to the alfalfa reference genome using hisat2 v4.8.2 [40], and Samtools was used to translate SAM file to BAM file and then to sort BAM files using default parameters [41]. FeatureCounts v2.0.1 was used to generate read counts for each sample [42].

### Leaf-specific genes retrieval

We used the R package TissueEnrich to identify leaf-specific genes [43]. The genes were divided into six groups based on their gene expression across the tissues in this package. In this research, leaf-specific expression genes include two groups (leaf-enriched and group-enriched). Leaf-enriched genes had an expression level in leaf tissue greater than or equal to 1 (FPKM), and also have at least five-fold higher expression levels in leaf tissue compared to all other tissues. Group-enriched genes with a FPKM value ≥ 1 that also have at least five-fold higher expression levels in leaf and another tissue compared to all other tissues, and that were not considered as leaf-enriched.

### Differentially expressed genes identification

DEGs, defined as |log2 fold change|≥ 5.259 and an adjusted *P* value of < 0.01, were screened using the R package DESeq2 [44].

### Potential candidate genes identification

Local blast search was conducted using TBtools software [45]. Briefly, based on the flanking marker sequences, the physical positions of the detected QTL were located on alfalfa genome (cultivar XingJiangDaYe) [22]. Within the physical region, sequences encoding protein products were extracted. Leaf-specific genes/DEGs within QTL intervals were annotated based on BLSATP search in NCBI (https://www.ncbi.nlm.nih.gov/) and Ensembl (https://ensembl.gramene.org/).

Quantitative real-time PCR (qRT-PCR) were used to determine whether candidate genes were differentially expressed in the two parents. qRT-PCR was conducted using unexpanded trifoliate leaves from three biological replicates of each parent. Six accessions with long leaf length and six accessions with short leaf length were selected to identify the relative expression levels of *MS.gene07851* in germplasms with different leaf length. These materials were grown in Langfang and leaf-related phenotypes were collected in 2018, 2019, and 2020. The information of these accessions was in placed in Supplementary Table 1. On a 7500 Real-Time PCR System (Applied Biosystems, CA, USA), qRT-PCR was implemented using the SYBR Premix Ex Taq (Takara, Japan). The relative gene expression level was calculated by the 2-△△Ct method.

In order to determine the relationship between polymorphic sites of the candidate genes and leaf development, variations were detected in each parent by comparing the resequencing data to the XingJiangDaYe reference genome. The process of obtaining BAM files for each genotype was consistent with RNA-seq data. GATK4.0 was used to perform variant calling [46], and the Heterozygous variants were filtered out. The resequencing data of the two parents have been deposited in the NCBI Sequence Read Archive under BioProject accession number PRJNA861857.

## Results

### Phenotypic analysis

**Fig. 1:**
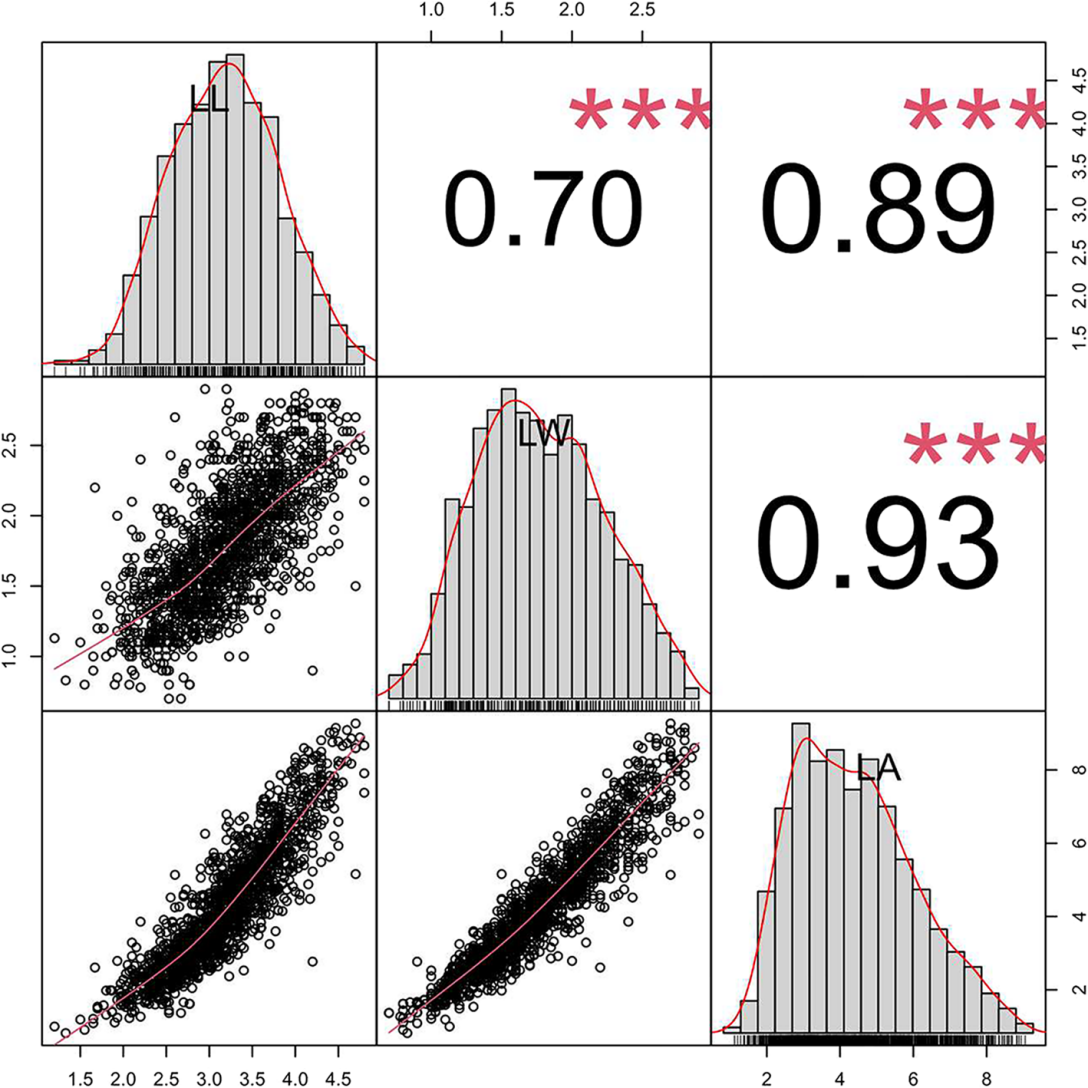
The phenotypic data (BLUP values) distribution and correlation coefficient between leaf-related traits in F_1_ population. *Above the diagonal* pearson’s correlation coefficient between LL, LW and LA traits. *Diagonal* represents the histogram of frequency distribution for each of the three traits in all environments. *Below the diagonal scatter* distribution between all traits

For F1 population and their parental plants, leaf size related traits including leaf length (LL), leaf width (LW), and leaf area (LA) in the tested environments were summarized. The average of leaf size of the paternal parent was significantly (*P* < 0.01) smaller than that of the maternal parent in the tested environments (Table 1). In the F1 population, three traits exhibited continuous distributions with transgressive segregations (Fig. 1, Table 1), suggesting that QTL analysis was feasible to dissect the leaf size components in alfalfa. The frequency distribution histogram of each trait generally conformed to a normal distribution in all environments (Fig. 1). Pearson’s correlation coefficient indicated highly significant (*P* < 0.001) degrees of correlation between two of the three traits at both locations (Fig. 1). The correlation coefficient was 0.93 between LA and LW, and 0.89 between LA and LL, suggesting that the LW contributes more to LA than LL does. The effects of genotype (G), environment (E) and their interaction (G x E) were highly significant (*P* < 0.001) for each trait analyzed. (Table S1). The average broad-sense heritability (*H*^2^) for LL, LW, and LA was 60%, 63%, and 64%, respectively, indicating that genetic factors played an important role in determining these traits (Table S1).

**Table 1:** Summary statistics analysis of phenotypes for LL, LW and LA in the F1 progeny and parents for two years in two locations (CP and LF)

Environment	Trait	Mean of paternal parent	Mean of maternal parent	F1			
				Mean ± SD	Range	Skewness	kurtosis
CP18	LL	2.37	4.30**	3.30 ± 0.02	1.40—5.00	-0.16	0.3
CP18	LW	1.2	2.17**	1.72 ± 0.01	0.90—2.90	0.47	-0.07
CP18	LA	2.07	6.76**	4.19 ± 0.05	1.19—8.26	0.42	-0.18
CP19	LL	2.47	4.00**	2.60 ± 0.02	0.20—4.70	-0.05	1.41
CP19	LW	1.17	1.73**	1.34 ± 0.01	0.90—2.90	0.74	1.21
CP19	LA	2.11	5.08**	2.58 ± 0.04	0.38—7.87	0.87	1.5
LF18	LA	2.6	7.99**	5.83 ± 0.07	0.66—9.76	-0.25	-0.18
LF18	LL	2.73	4.20**	3.64 ± 0.03	1.00—4.90	-0.51	0.63
LF18	LW	1.3	2.57**	2.16 ± 0.02	0.90—2.90	-0.55	0.46
LF19	LL	2.63	4.47**	3.14 ± 0.02	1.20—4.90	-0.12	-0.1
LF19	LW	1.3	2.07**	1.82 ± 0.01	0.90—2.90	-0.03	-0.19
LF19	LA	2.53	6.76**	4.31 ± 0.05	0.66—10.04	0.41	-0.18

All the data is the average of the phenotypes in each environment.

*LL* leaf length, *LW* leaf width, *LA* leaf area, *CP* Changping, *LF* Langfang Asterisks indicate significant differences between parents(t-test, *P* < 0.01)

### QTL analysis of leaf-related traits

Within the 32 homologs of the 8 alfalfa chromosomes, we detected 24 significant (*P* ≤ 0.05) QTL for leaf-related traits on both male and female linkage maps (Table 2). P1 contributed 4 of the 24 QTL, and 20 QTL were contributed by P2. Thirteen QTL were identified at the location of CP, seven at the location of LF, and four QTL were identified using BLUPs. These QTL contributed between 2.37%-22.45% of the phenotypic variance at different locations.

**Table 2:** QTL for leaf-related traits in an F1 population. The phenotypic data was assessed for two years in two locations (CP and LF)

Phenotype	Parent	QTL	Chr	Environment	Position	LSI (cM)	Flanking markers	LOD	PVE (%)	Add
LL	P2	*qLL-2B*	2B	CP18	41	40.5–42.5	TP47199-TP94214	4.44	5.38	-0.11
	P2	*qLL-6D-1*a	6D	CP18	40	39.5–40.5	TP87775-TP35294	4.92	6.09	0.12
	P2	*qLL-6D-2*a	6D	CP19	40	38.5–40.5	TP87775-TP35294	5.23	3.95	0.12
	P2	*qLL-6D-3*a	6D	BLUP	40	39.5–40.5	TP87775-TP35294	5.22	6.88	0.1
LW	P2	*qLW-2C*	2C	LF19	61	59.5–62.5	TP77362-TP23382	3.97	6.08	-0.08
	P2	*qLW-6B*	6B	LF19	24	23.5–24.5	TP66725-TP11462	4.77	7.91	-0.1
	P2	*qLW-6D-1*	6D	LF18	14	13.5–15.5	TP61730-TP2500	6.06	11.92	0.11
	P2	*qLW-6D-2*b	6D	CP18	27	26.5–28.5	TP53281-TP57492	18.87	22.45	0.14
	P2	*qLW-6D-3*c	6D	CP19	28	27.5–28.5	TP57492-TP29932	10.8	11.5	0.1
	P2	*qLW-6D-4*c	6D	BLUP	28	26.5–28.5	TP57492-TP29932	17.77	22.41	0.11
	P2	*qLW-6D-5*d	6D	LF18	34	32.5–35.5	TP24250-TP97699	6.39	12.73	0.11
	P1	*qLW-7D*	7D	LF19	57	56.5–57.5	TP55339-TP53717	6.09	9.19	-0.11
	P1	*qLW-8A*	8A	CP18	49	46.5–50.5	TP68418-TP59234	5.5	2.37	-0.07
	P2	*qLW-8B*	8B	CP18	89	87.5–90.5	TP88178-TP51967	4.15	4.27	-0.06
LA	P1	*qLA-4A*	4A	CP18	158	154.5–161.5	TP13527-TP67107	4.61	5.35	-0.29
	P2	*qLA-6D-1*b	6D	LF19	27	26.5–28.5	TP53281-TP57492	4.89	9.13	0.41
	P2	*qLA-6D-2*b	6D	BLUP	27	26.5–28.5	TP53281-TP57492	13.77	18.14	0.36
	P2	*qLA-6D-3*c	6D	CP19	28	27.5–28.5	TP57492-TP29932	11.08	9.69	0.28
	P2	*qLA-6D-4*d	6D	LF18	34	32.5–34.5	TP24250-TP97699	9.85	15.17	0.59
	P2	*qLA-6D-5*	6D	CP18	39	38.5–40.5	TP82945-TP87775	21.52	18.88	0.57
	P1	*qLA-7D-1*	7D	BLUP	49	48.5–49.5	TP26299-TP62590	15.54	18.14	-0.39
	P2	*qLA-7D-2*	7D	CP19	45	43.5–46.5	TP49287-TP39017	3.87	3.49	-0.17
	P2	*qLA-7D-3*	7D	CP19	90	88.5–90.5	TP32383-TP53416	4.62	4.1	0.18
	P2	*qLA-8C*	8C	CP18	126	125.5–126.5	TP82035-TP43195	5.65	3.97	0.27

QTL, q + trait abbreviation + chromosome + QTL number, e.g., qLL-6D-1, corresponds to the first QTL for LL on chromosome 6D; CP and LF refer to the two locations—Changping and Langfang, respectively; Chr, Chromosome; LSI 1-LOD support interval in cM unit; Flanking markers interval of the QTL; LOD Logarithm of odds for each QTL; PVE, the percentage of the phenotypic variation explained by QTL; Add, the additive effects of the QTL. QTLs at the same genetic position were marked with the same lowercase letter; QTLs with PVE > 10% were underlined

*QTL associated with leaf length*. Four significant QTL (*qLL-2B*, *qLL-6D-1*, *qLL-6D-2*, and *qLL-6D-3*) associated with LL were mapped on the maternal linkage maps. The QTL were detected on linkage group (LG) 2B, and 6D (Figs. S1 and S2). The phenotypic variation explained (PVE) by a single QTL ranged from 3.95% to 6.88% in different environments (Table 2). Among them, the QTL (*qLL-2B*) was on LG 2B at the position of 40.5–42.5 cM with PVE value of 5.38%. The rest three QTL (*qLL-6D-1*, *qLL-6D-2*, and *qLL-6D-3*), located on LG 6D of P2 from the 38.5 cM to 40.5 cM position, were considered to be the same QTL.

*QTL associated with leaf width*. Ten QTL for LW were located on homologs 2C (1 QTL), 6B (1 QTL), 6D (5 QTL), 7D (1 QTL), 8A (1 QTL), and 8B (1 QTL) with an individual QTL accounting for 2.37–22.45% of phenotypic variations (Table 2, Figs. S1 and S2). Among them, two QTL were identified in P1 and 8 QTL in P2 with five QTL (*qLW-6D-1*, *qLW-6D-2*, *qLW-6D-3*, *qLW-6D-4*, and *qLW-6D-5*) located on chromosome 6D. These five QTL explained 11.50%-22.45% of the phenotypic variation for LW.

*QTL associated with leaf area*. For LA, 10 significant QTL were identified on LG 4A, 6D, 7D, and 8C. These QTL explained 3.49 to 18.88% of the phenotypic variation in different environments (Table 2, Figs. S1 and S2). The LSI (1-LOD support interval in cM unit) of these QTL was from 1 to 7 cM. Five QTL (*qLA-6D-1*, *qLA-6D-2*, *qLA-6D-3*, *qLA-6D-4*, and *qLA-6D-5*) were co-localized on chromosome 6D in P2 with higher phenotypic variation than the most other QTL explained averagely for 7.01% for LA. Among them, the QTL *qLA-6D-5* explained the highest (18.88%) phenotypic variance for LA, implying a higher reliability of the loci on 6D for leaf area in alfalfa.

*Overlapped QTL intervals*. Four overlapped QTL intervals, which were identified at the same position in different environments/phenotype, were detected. All overlapped intervals were identified in P2 parent on chromosome 6D. *qLL-6D-1*, *qLL-6D-2*, and *qLL-6D-3* located in 38.5–40.5 cM on chromosome 6D. *qLW-6D-2* and *qLA-6D-1* were identified at CP and LF, respectively. And they were overlapped with *qLA-6D-2*. *qLW-6D-3*, *qLW-6D-4*, and *qLA-6D-3* were located at the position of 28 cM with an average PVE value of 14.53%. The last overlapped interval (*qLW-6D-5* and *qLA-6D-4*) was at the position of 34 cM with an average PVE value of 13.95%.

We focused on six major QTL intervals, including four overlapped QTL intervals and two QTLs with high PVE values (≥ 10%). The physical interval of the co-located QTL, including *qLW-6D-5* and *qLA-6D-4*, was really large (25.1 ~ 74.6 Mb). So we selected ± 2 Mb physical intervals of two flanking markers to predict candidate genes. Based on local-BLAST, a total of 1,573 genes were identified in seven physical regions, which were used for further analysis in our study.

### Leaf-specific genes within QTL regions

**Fig. 2:**
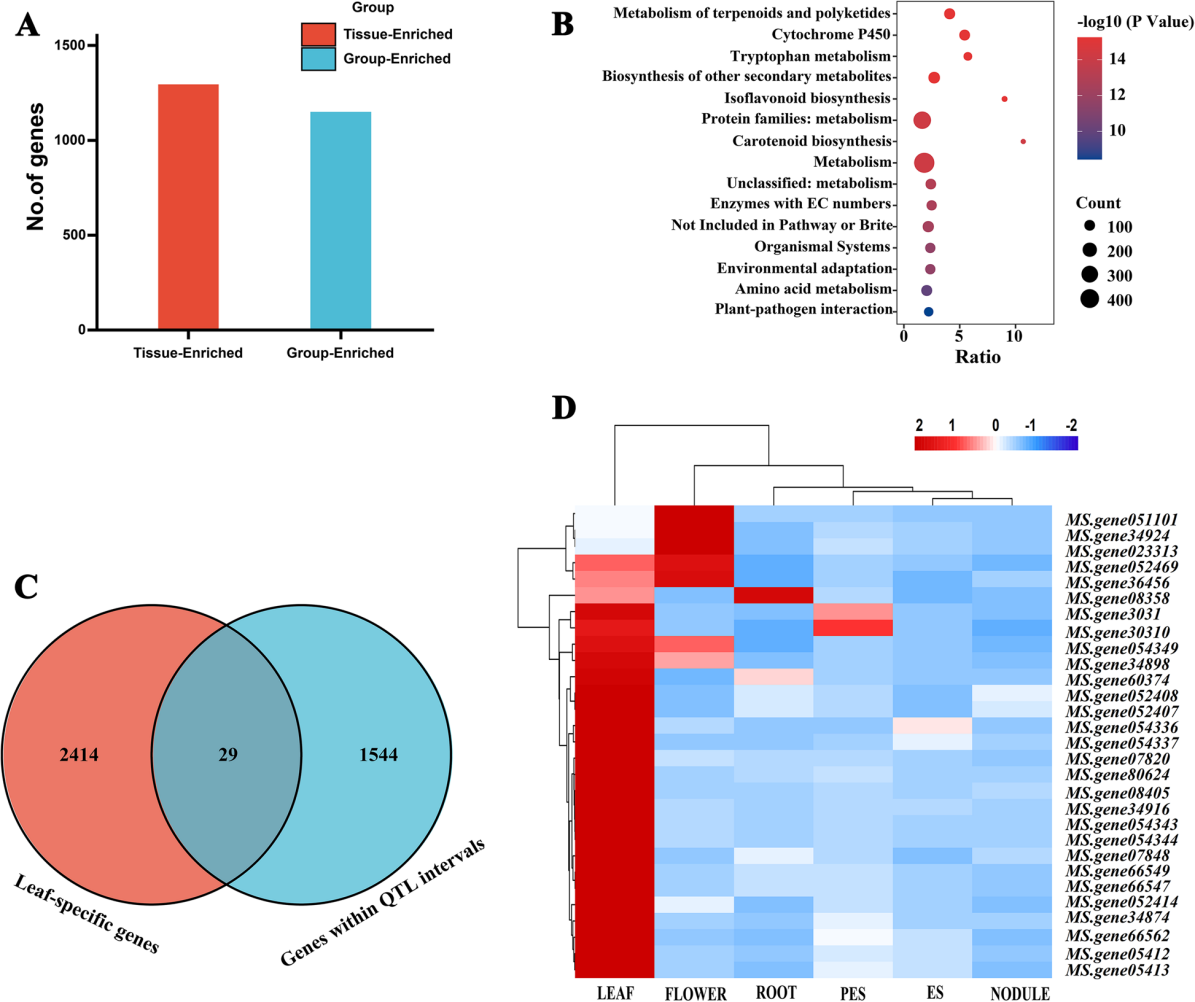
Leaf-specific genes within QTL regions. **A** Number of leaf-enriched genes (red) and group-enriched genes (blue) in six tissues. Tissue-Enriched genes, expression level in leaf tissue ≥ 1 (FPKM) and also have at least five-fold higher expression levels in leaf tissue compared to all other tissues; Group-Enriched genes, expression level in leaf tissue ≥ 1 (FPKM), have at least five-fold higher expression levels in leaf and another tissue compared to all other tissues, and that were not considered as Tissue-Enriched genes. **B** Bubble chart of KEGG enrichment analysis of the leaf-specific genes in the six tissues (www.kegg.jp/kegg/kegg1.html). The abscissa represents enrichment factor and the ordinate represents different KEGG terms. Circle size represents the gene number while circle color represents the value of − log10 (*p*). **C** Venn diagram of leaf-specific genes and genes within QTL intervals. (**D**) Heatmap clustering the 29 common genes by their expression abundance

**Fig. 3:**
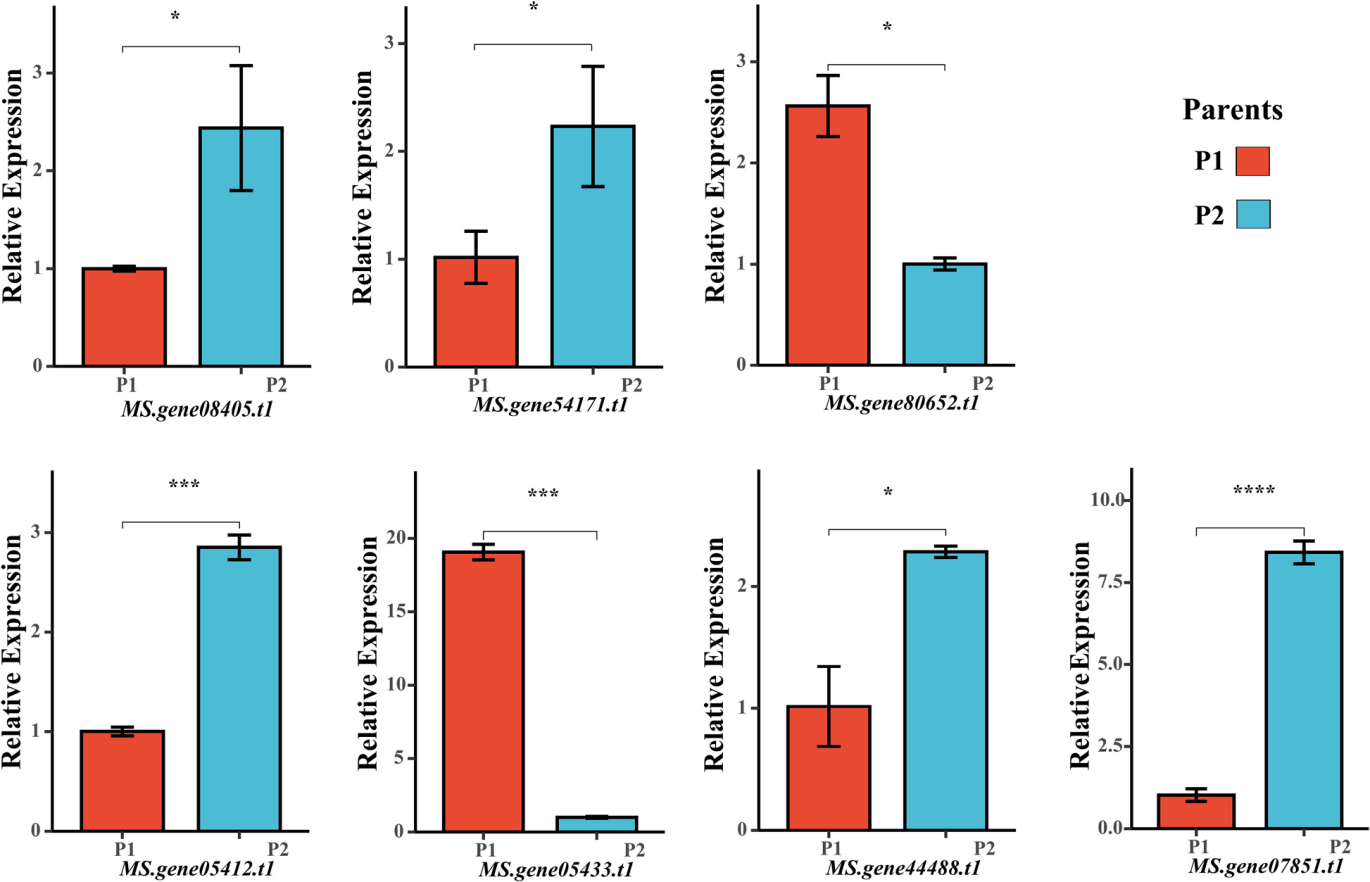
qRT-PCR analysis of the seven candidate genes in young leaves in two parents. Asterisk symbols indicate significant differences between two parents (t-test, *, *P* < 0.05; ***, *P* < 0.001, ****, *P* < 0.0001)

A total of 2,443 leaf-specific genes were identified, including 1,294 leaf-enriched and 1,149 group-enriched (Fig. 2A). Among the significantly KEGG enriched pathways, metabolism and biosynthesis pathway contained more genes than the others, indicating that these metabolism and biosynthesis pathway might have a modulating effect on the function and development of alfalfa leaves (Fig. 2B). Leaf-specific analysis and QTL mapping revealed 29 common genes, and four of them were predicted as candidates based on BLAST-P (Fig. 2C and 2D). These genes are involved in cell proliferation and growth, and their homologous have been reported to play a role in leaf development. The expression levels of these four genes were further investigated in the young leaves of the two parents. The result showed that *MS.gene08405* and *MS.gene05412* were differently expressed in the two parents (Fig. 3). They were located in two different QTL intervals of *qLA-7D-1* (*MS.gene08405*), *qLW-6D-5* and *qLA-6D-4* (*MS.gene05412*) (Table S5).

### DEGs within QTL regions

**Fig. 4:**
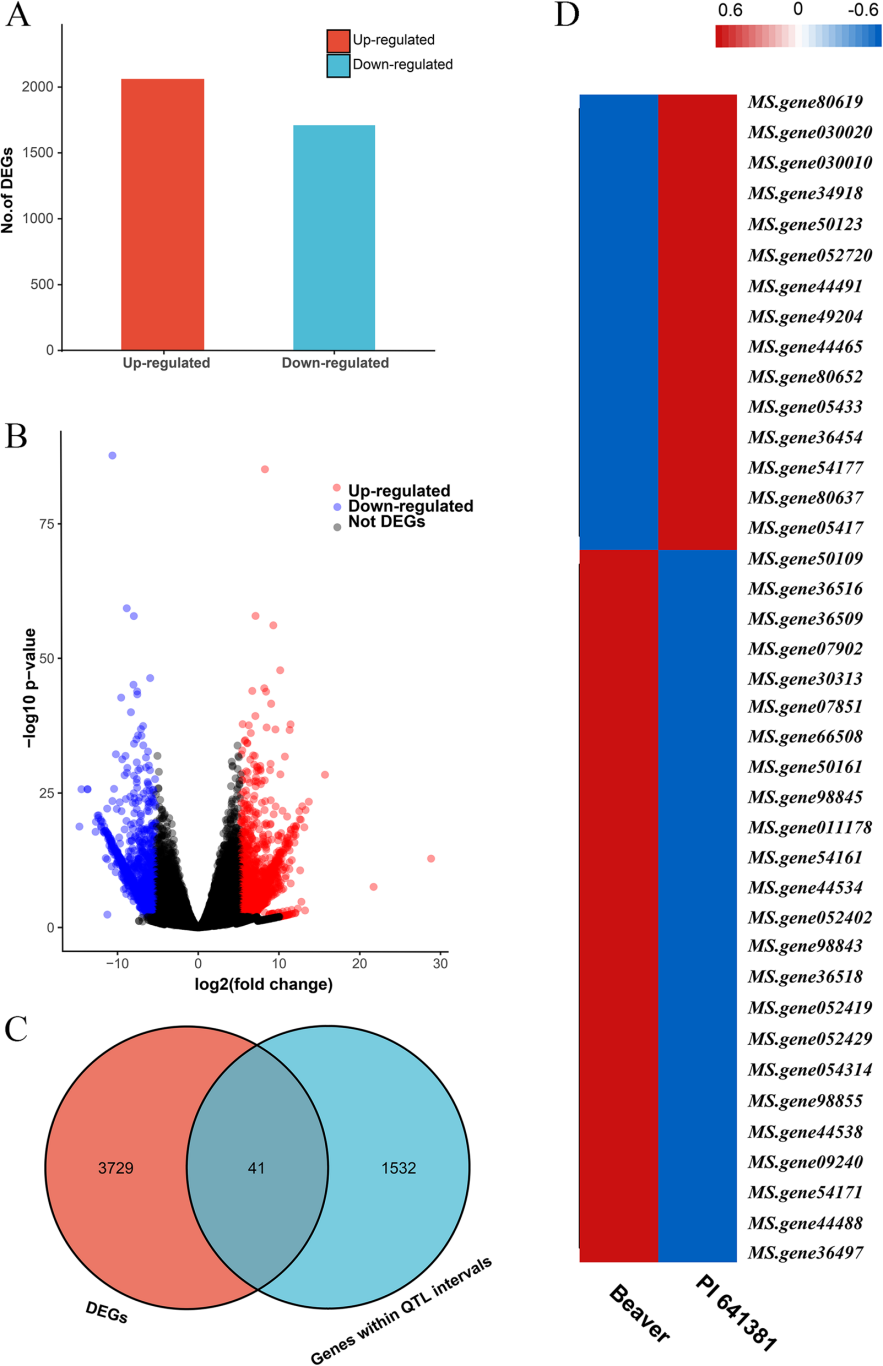
Differentially expressed genes (‘*sativa*’ Vs ‘*falcata*’) within QTL intervals. **A** Number of up-regulated genes (red) and down-regulated genes (blue). **B** Volcano plot of DEGs. Up- and down-regulated genes are reported as red and blue dots, respectively; not DEGs are represented as gray dots. **C** Venn diagram of DEGs and genes within QTL intervals. **D** Heatmap clustering the 41 common genes by their expression abundance

**Fig. 5:**
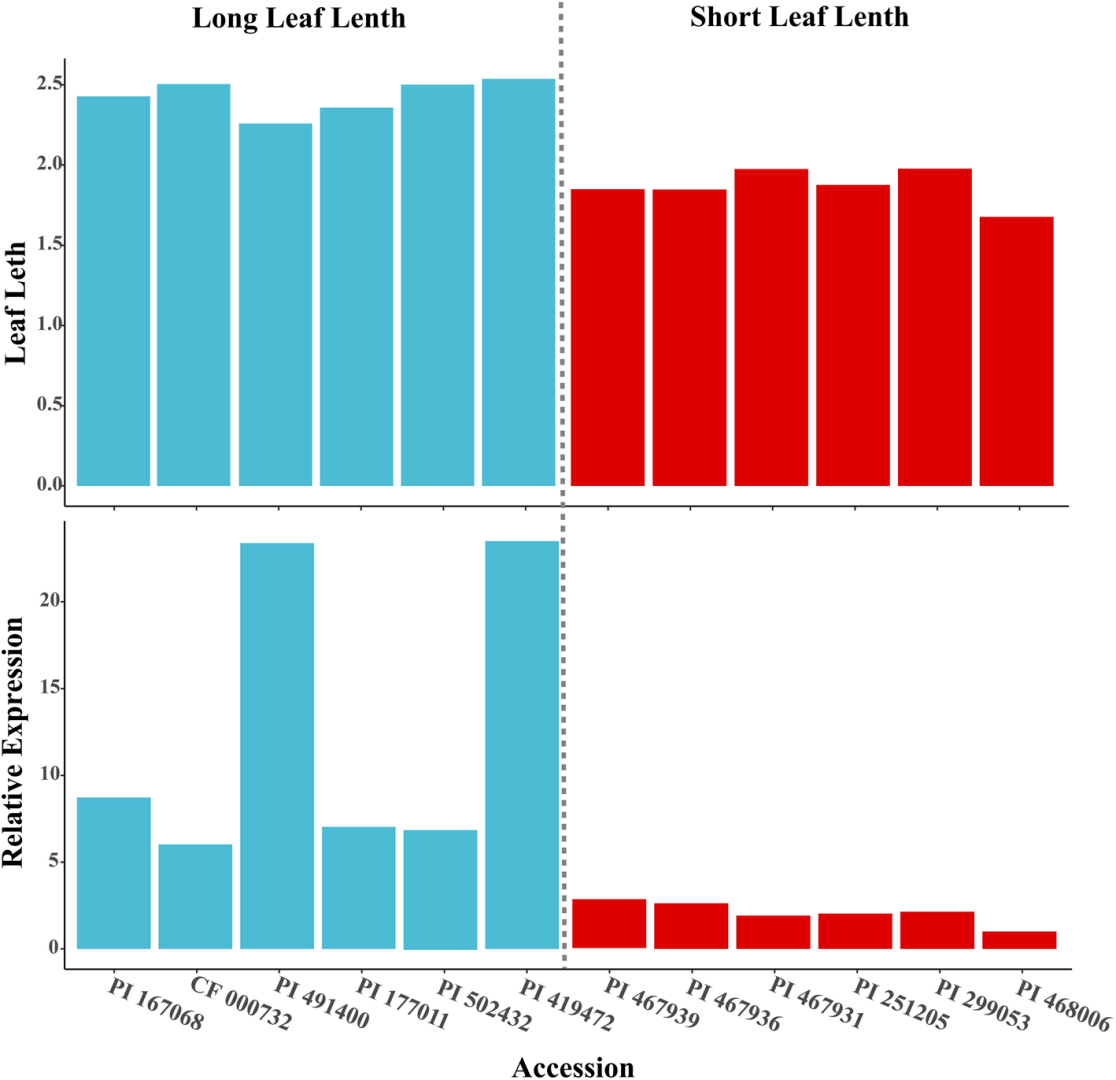
Expression of *MS.gene07851* in young leaves of 12 accessions. **A** Leaf length of the 12 accessions. Date were the mean of three years in LangFang. **B** Relative expression of *MS.gene07851*

Compared to ‘*sativa*’, leaf size was 43.3% relative decrease in ‘*falcata*’. DEG analysis identified 2,061 up-regulated and 1,709 down-regulated DEGs (‘*sativa*’ Vs ‘*falcata*’) in leaf tissue of the two genotype (Fig. 4A and 4B; Table S3; Table S4). A total of 41 DEGs located within the QTL physical intervals (Fig. 4C). And nine DEGs, whose homologous genes were reported to have functions in cell proliferation and cell expansion, were annotated as candidates associated with leaf development in plants. Among them, five genes, which had different expression patterns in two parents, were identified as candidate genes (Fig. 3). The five candidates were located in four intervals, including: *qLW-6D-1* (*MS.gene54171* and *MS.gene80652*), *qLW-6D-5*, *qLA-6D-4* (*MS.gene05433*), *qLW-6D-3*, *qLW-6D-4*, and *qLA-6D-3* (*MS.gene44488*), *qLL-6D-1*, *qLL-6D-2*, *qLL-6D-3* (*MS.gene07851*) (Table S5). *MS.gene07851*, which were located in three LL-related QTLs, had a significant expression difference (*P* < 0.0001) between the two parents. In addition, long leaf length accessions displayed high expression levels of *MS.gene07851*, which was in accordance with their LL values (Fig. 5). Thus, *MS.gene07851* might be the causal gene that underlies variation in the leaf length.

### Variation of candidate genes between the parental genotypes

We investigated the polymorphic sites among the seven candidate genes by comparing their whole-genome re-sequencing reads with the reference genome. By mapping to the XingJiangDaYe genome, 26 SNPs and 3 InDels were detected among the seven candidate genes between two genotypes (Table S5). Among them, five SNPs caused amino acid changes in three candidate genes of *MS.gene05433* (three), *MS.gene44488* (one), and *MS.gene07851.t1* (one). Notably, the gene *MS.gene07851* showed a SNP (C/G) variant in the two parental genotypes, which may correspond to its different function in leaf development. These results revealed multiple variants in the seven candidate genes, suggesting that the variants may contribute to different protein functions and differences in leaf size between the two parents.

## Discussion

Leaf size is an important target to increase yield in plant breeding because it affects crop canopy morphology and photosynthetic efficiency [47]. For forage crop, alfalfa, increasing leaf size is an important approach to improve its yield and quality. However, the genetic mechanisms of alfalfa are intricate due to its homologous polyploidy, cross-pollinated, and highly heterozygous in nature [22]. To date, there have been less reports regarding leaf development genes in alfalfa.

RNA-sequencing (RNA-seq), a high resolution technology, can provide far more massive amounts of information regarding the gene expression levels of various species in different conditions [48, 49]. Based on quantifications of expressed genes, differentially expressed genes are a group of genes which have a statistically significant difference at the expression level. DEG analyses can provide reliable insight into the genetic mechanisms in crops that have different phenotype, including plant at different development stages and in different growing conditions [50, 51]. The tissue-specific genes refer to genes specifically expressed in one or several tissues/cell types, which involve regulate tissues-specific morphological structures or physiological functions. Identification of these genes can help us better understanding of tissue-gene relationship. In alfalfa, RNA-seq analysis uncovered molecular mechanisms underlying abiotic stress, including salt [52, 53], drought [54, 55], and cold tolerance [56]. Meanwhile, pathways involved in early flowering [57] and differentially expressed genes related to defoliation traits [58] had also been identified in alfalfa by transcriptome analysis. In the mapping population, the two parents not only had a significant difference in leaf size but also in other traits such as plant height and yield [59]. ‘Beaver’ and ‘PI 641,381’, appeared morphologically similar overall, and no significant differences were noted between genotypes in terms of plant height, aboveground biomass, or root length. ‘PI 641,381’ had a significant reduction in leaf area compared to 'Beaver' [37].

QTL mapping, a powerful method to identify trait associated QTLs/genes, has been reports on alfalfa important agronomic traits [25, 26, 60]. Here, we mapped 24 QTL for leaf size using the high density genetic maps. The effects of environment were highly significant (*P* < 0.001) for each trait. No QTL was identified at the same genetic position by using the data from different cultivated condition. Most of the detected QTLs for LA, LL and LW were different. Only three QTL intervals were detected for both LW and LA (Table 2). In this study, the genetic linkage maps were constructed by GBS-SNP markers. Although, using GBS-SNP mapping greatly facilitates genetic dissection of quantitative traits in alfalfa. There are some limitations of this strategy. GBS missed a lot of genotypic information, and many polymorphic markers not classified as SDA were not used for map construction, resulting in reduced power of QTL detection [23].

The traditional QTL fine mapping and map-based cloning in alfalfa are limited by constructing near-isogenic lines. So far, QTL mapping studies related to alfalfa important agronomic traits were mainly focused on the QTL identification [61]. Few studies have made an effort to identify candidate genes within the QTL ranges. Transcriptome analysis can be used to characterize gene expression levels in various species. In recent years, RNA-seq has been used in QTL mapping studies to rapidly identify potential candidate genes in crops [30, 31, 62]. The strategy of QTL mapping combined with RNA-seq allowed for rapid detection of candidate genes in target regions. A total of seven candidates associated with leaf development were finally detected in our study.

The processes that mainly drive leaf development are cell proliferation and cell expansion, and numerous genes have been identified in Arabidopsis, such as *CYP78A5*, *DA1*, and *MYBH* [7, 63, 64]. *CYP78A5*, a member of cytochrome P450 protein family, which played an important role in cell proliferation and expansion [65], was a homologous gene of one candidate (*MS.gene08405*). It was annotated with the function of stimulating cell proliferation during leaf development [64]. Interestingly, *MS.gene07851* was annotated as protein tyrosine and serine/threonine kinase, which contained conserved domain PK_Tyr_Ser-Thr. Protein kinase has conserved function in several cellular processes, including division, proliferation, apoptosis, and differentiation [66–68]. The family members have been reported to regulate leaf development in Arabidopsis [68, 69]. SNPs are the major determinant of phenotypic differences [70]. Thus, we can hypothesize that the identified SNP in *MS.gene07851*, may affect their expression and function. At the same time, the results of qRT-PCR showed that the expression levels of *MS.gene07851* in long-leaf germplasms were higher than that in short-leaf germplasms, indicating that it may be involved in the regulation of leaf length development. The candidate genes identified in our study should be confirmed by genetic transformation and further studies.

In summary, QTL mapping and transcriptome analysis were performed to identify the genes for leaf development in an F1 population derived from a larger leaf area cultivar named Zhong mu No.1 and a smaller leaf area accession named Cangzhou. Six important QTL regions were identified, which contained seven candidate genes. This study provides valuable resources for enhancing breeding programs aimed at improvement of leaf development in alfalfa.

## Supplementary Information

**Additional file 1: Fig. S1** Leaf- related QTLs on 32 linkage groups from a genetic linkage map of paternal parent (P1).

**Additional file 2: Fig. S2** Leaf- related QTLs on 32 linkage groups from a genetic linkage map of maternal parent (P2).

**Additional file 3: Table S1** Analysis of variance for LL, LW and LA in an F1 population using mixed model for two years in two locations (CP and LF)

**Additional file 4: Table S2.** List of 2,443 leaf-specific genes.

**Additional file 5: Table S3. **List of 2,061 up-regulated genes (‘*sativa*’ Vs ‘*falcata*’).

**Additional file 6: Table S4.** List of 1,709 down-regulated genes (‘sativa’ Vs ‘falcata’).

**Additional file 7: Table S5.** The information of the seven candidates.

## Data Availability

The raw data of GBS were submitted to the NCBI Sequence Read Archive with bioproject ID: PRJNA522887 (https://www.ncbi.nlm.nih.gov/bioproject/?term=PRJNA522887). RNA-seq raw data used in this study was downloaded from the SRA database in NCBI with the Bioproject accession numbers of PRJNA765383 (https://www.ncbi.nlm.nih.gov/bioproject/PRJNA765383) and PRJNA276155 (https://www.ncbi.nlm.nih.gov/bioproject/PRJNA276155). The Resequencing data have been deposited in the NCBI with the BioProject accession number PRJNA861857 (https://www.ncbi.nlm.nih.gov/bioproject/PRJNA861857).

## Abbreviations

ADD: Additive values
BLUP: Best linear unbiased predictions
cM: Centimorgan
DEGs: Differentially expressed genes
GBS: Genotyping by sequencing
G x E: Genotype by environment
H2: Broad-sense heritability
ICIM-ADD: Composite interval mapping with additive effect
LA: Leaf area
LG: Linkage group
LL: Leaf length
LOD: Logarithm of odds for each QTL
LW: Leaf width
PVE: Phenotypic variation explained
MAS: Marker-assisted selection
QTL: Quantitative trait loci
RNA-seq: RNA-sequencing
SDA: Single dose allele
SNP: Single nucleotide polymorphism
