# Supplementary figures and images for: Combining QTL mapping and RNA-Seq Unravels candidate genes for Alfalfa (Medicago sativa L.) leaf development
Source: BMC Plant Biol. 2022 Oct 11;22:485. doi: 10.1186/s12870-022-03864-7 (PMC9552516; doi:10.1186/s12870-022-03864-7)

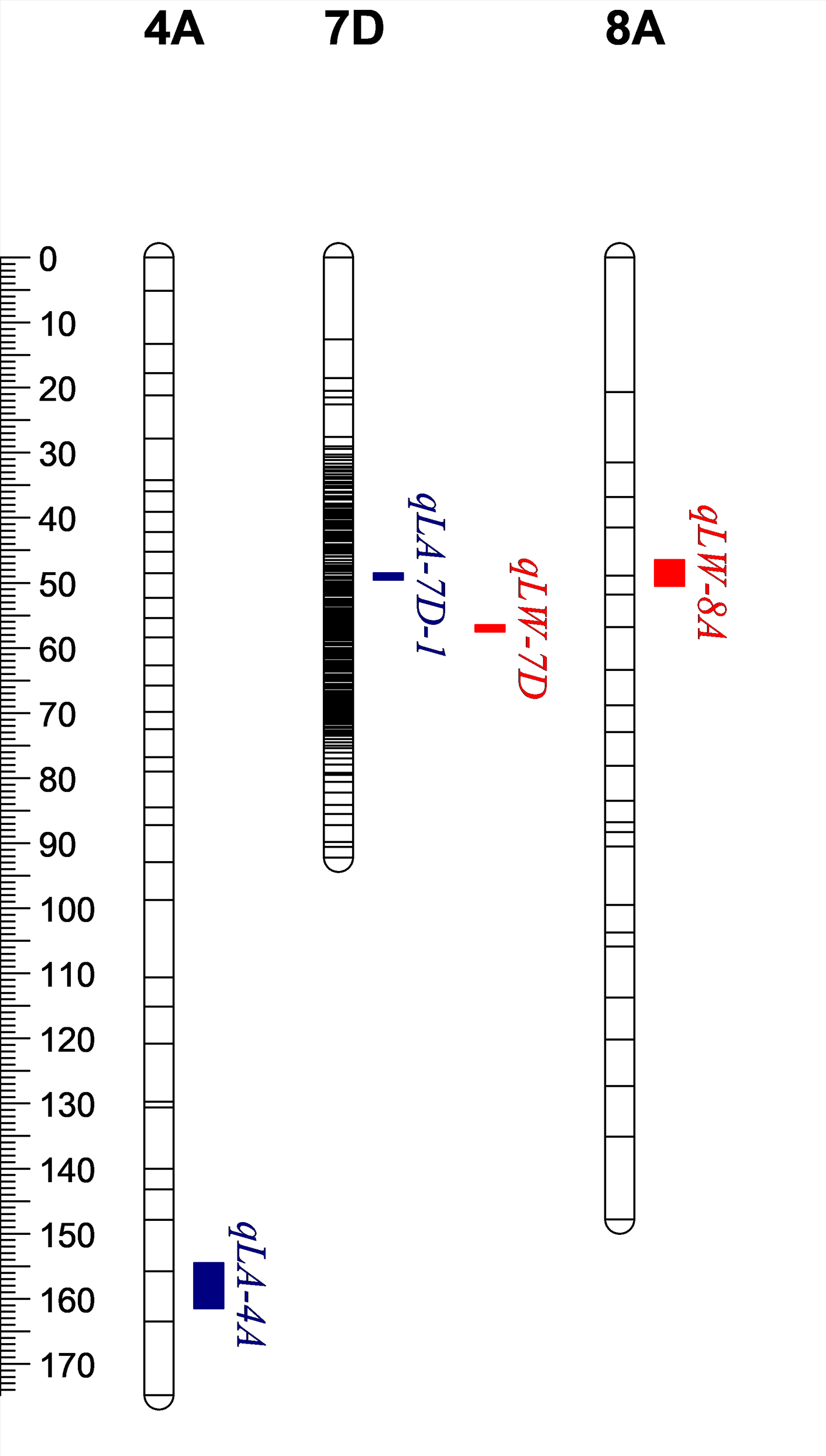

Supplement: Supplementary file 1 — Additional file 1: Fig. S1 Leaf- related QTLs on 32 linkage groups from a genetic linkage map of paternal parent (P1). [file 12870_2022_3864_MOESM1_ESM.tif]

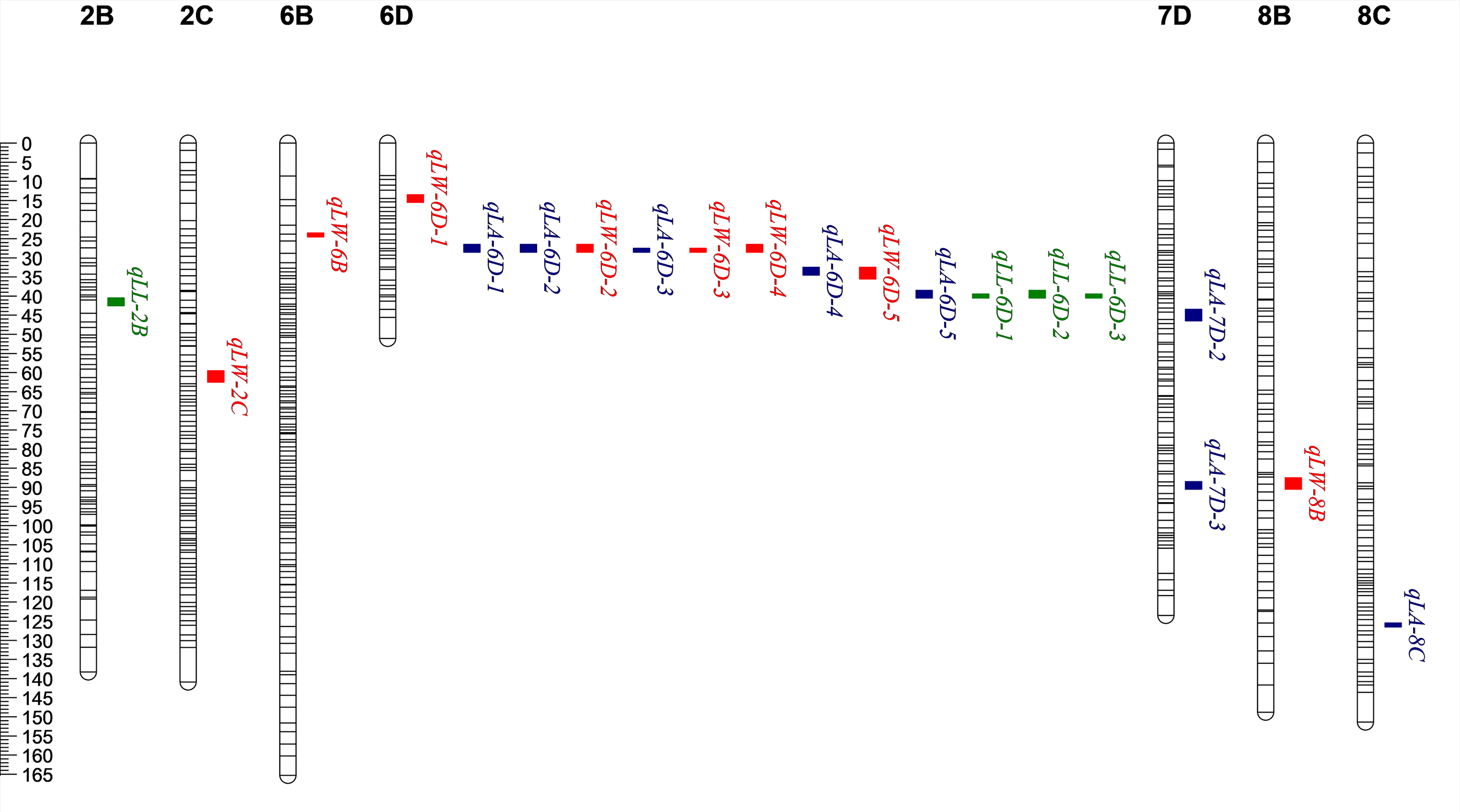

Supplement: Supplementary file 2 — Additional file 2: Fig. S2 Leaf- related QTLs on 32 linkage groups from a genetic linkage map of maternal parent (P2). [file 12870_2022_3864_MOESM2_ESM.tif]
